# Combining microfluidics with machine learning algorithms for RBC classification in rare hereditary hemolytic anemia

**DOI:** 10.1038/s41598-021-92747-2

**Published:** 2021-06-30

**Authors:** Valeria Rizzuto, Arianna Mencattini, Begoña Álvarez-González, Davide Di Giuseppe, Eugenio Martinelli, David Beneitez-Pastor, Maria del Mar Mañú-Pereira, Maria José Lopez-Martinez, Josep Samitier

**Affiliations:** 1 Josep Carreras Leukaemia Research Institute (IJC), 08916 Badalona, Spain; 2 Institute for Bioengineering of Catalonia (IBEC), Barcelona Institute of Science and Technology BIST, 08028 Barcelona, Spain; 3 Department of Medicine, University of Barcelona, 08036 Barcelona, Spain; 4 Department of Electronic Engineering, University of Rome Tor Vergata, 00133 Rome, Italy; 5 University of Rome Tor Vergata, Interdisciplinary Center for Advanced Studies on Lab-on-Chip and Organ-on-Chip Applications (ICLOC), 00133 Rome, Italy; 6 Centro de Investigacion Biomedica en Red en Bioingeniería, Biomateriales y Nanomedicina (CIBER-BBN), 28029 Madrid, Spain; 7 Red Blood Cell Disorders Unit, Hematology Department, Vall d’Hebron University Hospital, Vall d’Hebron Institute of Oncology (VHIO), 08035 Barcelona, Spain; 8 Rare Anemia Disorders Research line, Translational Research in Child and Adolescent Cancer, Vall d’Hebron Research Institute (VHIR), ERN-EuroBloodNet Member, 08035 Barcelona, Spain; 9 Department of Electronic and Biomedical Engineering, University of Barcelona, 08028 Barcelona, Spain

**Keywords:** Anaemia, Sickle cell disease, Microfluidics, Machine learning

## Abstract

Combining microfluidics technology with machine learning represents an innovative approach to conduct massive quantitative cell behavior study and implement smart decision-making systems in support of clinical diagnostics. The spleen plays a key-role in rare hereditary hemolytic anemia (RHHA), being the organ responsible for the premature removal of defective red blood cells (RBCs). The goal is to adapt the physiological spleen filtering strategy for in vitro study and monitoring of blood diseases through RBCs shape analysis. Then, a microfluidic device mimicking the slits of the spleen red pulp area and video data analysis are combined for the characterization of RBCs in RHHA. This microfluidic unit is designed to evaluate RBC deformability by maintaining them fixed in planar orientation, allowing the visual inspection of RBC’s capacity to restore their original shape after crossing microconstrictions. Then, two cooperative learning approaches are used for the analysis: the majority voting scheme, in which the most voted label for all the cell images is the class assigned to the entire video; and the maximum sum of scores to decide the maximally scored class to assign. The proposed platform shows the capability to discriminate healthy controls and patients with an average efficiency of 91%, but also to distinguish between RHHA subtypes, with an efficiency of 82%.

## Introduction

The spleen is the organ specialized in filtering the blood, removing old and defective red blood cells (RBCs). This filtering function is specifically performed by the red pulp, where the blood is forced to flow through Inter Endothelial Slits (IES), whose diameter can reach 1 μm^1,2^.

Human RBCs are 120-day lifespan cells, known for containing hemoglobin (Hb), necessary to accomplish their main function as oxygen transporters. They have a diameter and thickness of 8 and 2–3 μm, respectively, presenting a unique flexibility characteristic needed to rapidly deform themselves and squeeze into the blood flow; particularly into the capillaries which diameter can reach half of the size of an RBC^3,4^.

Defects affecting RBCs can lead to rare hereditary hemolytic anemia (RHHA), a group of heterogeneous inherited disorders characterized by premature removal of RBCs (hemolysis) in the spleen, as they are not able to pass through the IES. Consequently, Hb level concentration decreases, sometimes insufficient to fulfill physiology needs^5^. RHHA RBCs can show changes in volume, plasticity, shape and deformability leading to compromised mechanical behavior.

Depending on the affected RBC’s component, RHHAs are classified as: (i) hemoglobinopathies, which include sickle cell disease (SCD) and thalassemia syndromes (THAL), (ii) membranopathies, as hereditary spherocytosis (HS), and (iii) enzymopathies. SCD is characterized by a single mutation in the gene encoding the Hb subunit β (HBB) responsible for the formation of a structurally abnormal Hb variant, known as HbS (sickle Hb)^6^. Indeed, under deoxygenated conditions, HbS molecules adhere in large quantities, forming Hb polymers which precipitates and that deform the structure of the RBC, referred to as ‘sickling. HbS polymerization changes shape and physical properties of RBC interfering with their flexibility and rheological properties. Repeated episodes of HbS polymerization and RBC sickling in conditions of low pO_2_ and unsickling in conditions of high pO_2_ can lead to severe alterations in the membrane structure and function and eventually result in the formation of an irreversibly sickle cell. Deformed sickle RBCs are rigid, interact with leucocytes, platelets and the vascular endothelium and can occlude blood flow in the microvascular circulation producing vascular damage, organ infarcts, painful episodes and other symptoms^7–9^. Concerning HS, it is caused by mutations in genes encoding RBC membrane proteins, causing defects in the vertical interactions and leading to spherically-shaped RBCs with decreased deformability^10^. Finally, Thalassemia is characterized by genetic abnormalities responsible for the absence or the decrease of the synthesis of one or more globin chains^11^. The resulting denatured Hb molecules precipitate and attach to the RBC membrane forming the so-known Heinz bodies, described as a cause for malfunctioning and the altered deformability of the RBCs^3,12,13^. Besides the heterogeneity among patients in RHHAs pathophysiology, therapies have been traditionally non-specific, limited to symptomatic control of the anemia. Conventional techniques for monitoring patients are based on the quantitative or qualitative measurement of the different blood components, while other technologies, as ectacytometry or flow-cytometry, are not always implemented in the lab routine of patients monitoring, probably due to their high cost and not easy access^14^. Therefore, there is a need for finding more accessible and easy techniques for RHHA monitoring studies. Indeed, the ability to monitor the disease’s phenotype would open to the possibility of the development of new treatment strategies and could have potential in prediction of disease complications and patient’s response to therapy. Besides, techniques as ectacytometry, that gives a results as a mean value, use whole or diluted blood and do not consider samples’ heterogeneity and difference in size among RBCs^15^.

Microfluidics technologies have gained an interesting role in this scenario, offering simple, low cost and rapid platform able to mimic the microvasculature properties reflecting cellular/tissue level response. It represents a suitable and high-throughput single-RBC-based approach as it can give us the chance to study RBCs biomechanical properties, overcoming the limitation of representing microcirculatory stress given by static methods. Several devices able to test a huge number of RBCs and to mimic microcirculatory pattern have been described^15^. Furthermore, microfluidics devices constitute also a way to facilitate and speed the efficacy validation for monitoring^14^. Microfluidics and Lab-on-a-Chip (LoC) technology constitute a valid choice for the study of red blood cells characteristics and to mimic spleen filtering behavior, using precise manipulation of fluid dynamics^16^. Evaluation of the spleen function based on the characterization of RBC mechanical properties, and their observation, may play a key role in the prognosis and morbidity of RHHA patients. Several microfluidics devices have been developed for the study of RBCs deformability, considered as a valid mechanical parameter for disease diagnosis^17–24^. Faustino et al. developed a device for the estimation of single-cell deformability through a hyperbolic converging micro channels to measure deformation and cell motion from healthy and end-stage kidney disease patients^18^. Lizarralde et al. fabricated a microfluidic device to mimic the mechanical stress on flowing sickle RBCs, being able to evaluate their resistance to lysis^19^. This device was also adapted to use in a bioimpedance-based approach to evaluate RBCs elasticity by electrically measuring their transit time in pathological and healthy RBCs^24^. Mehri et al. created a device combined with optical viscometer techniques for sorting RBCs to simultaneously measure viscosity with shear rates and aggregates sizes, and capable of maintaining RBCs integrity during cold storage and so to improve the efficiency of transfusions^21^.

Some works are focused on the study of RBC morphology, by applying deep learning and shape feature extraction^25–28^. Among these, an automated convolutional neural network (CNN) based approach for the shape classification of flowing RBCs in microcapillary flow was described^29^. Devices designed for in flux measurement, usually combined microfluidic techniques with sophisticated equipment such as laser diffraction viscometer, laser scanner electron microscope, real-time deformability cytometry, sensors or supernatants measurements with the scope to evaluate RBC deformability properties on healthy donors or in presence of blood disorders^19,30^. Most of them considered a limited number of donors and the manual user-dependent analysis by means of commercial tools (e.g., ImageJ, Adobe Photoshop)^19,26,30,31^.

A new frontier for microfluidic devices appears when they meet machine learning algorithms for image analysis; aiming not only to simulate organ functionalities but also to conduct massive quantitative cell behavior analysis and implement smart decision-making systems in support to clinical diagnostic. The use of non-invasive acquisition techniques such as Time-Lapse Microscopy (TLM) allows label-free analysis (with no fluorescence). Then, one of the straightforward applications of such an approach is to adapt the physiological spleen filtering strategy for in vitro study and monitoring of blood diseases through Red Blood Cell shape analysis.

We propose a microfluidic platform that mimics the flow conditions of the spleen in the IES region, combined with image analysis based on deep learning algorithms for the massive and objective study of RBCs deformation properties with the aim to distinguish between specific types of RHHA, using for our study human blood samples from healthy donors and from patients (SCD, THAL, and HS).

Specifically, we assumed that RBCs have the capacity of restoring their original shape after crossing IES which could be used as a parameter to measure deformability, going far beyond the analysis of old and/or defective RBCs. Furthermore, the exploitation of the deep learning algorithms adds the chance to conduct user-independent and automatic analysis. The automatic localization of cells in the image, performed by the design of image analysis modules, compensates the label-free acquisition condition and prevents laborious manual cropping of each cell. TLM permits to implement a continuous monitoring using low-cost equipment for the acquisition, on the contrary of some of the previously mentioned costly devices. At present, to author’s knowledge, it does not exist a platform that combines in an automatic way microfluidic device with non-invasive Time-Lapse Microscopy (TLM), video and smart data analysis of flowing cells for RBC cells.

## Results

**Figure 1:**
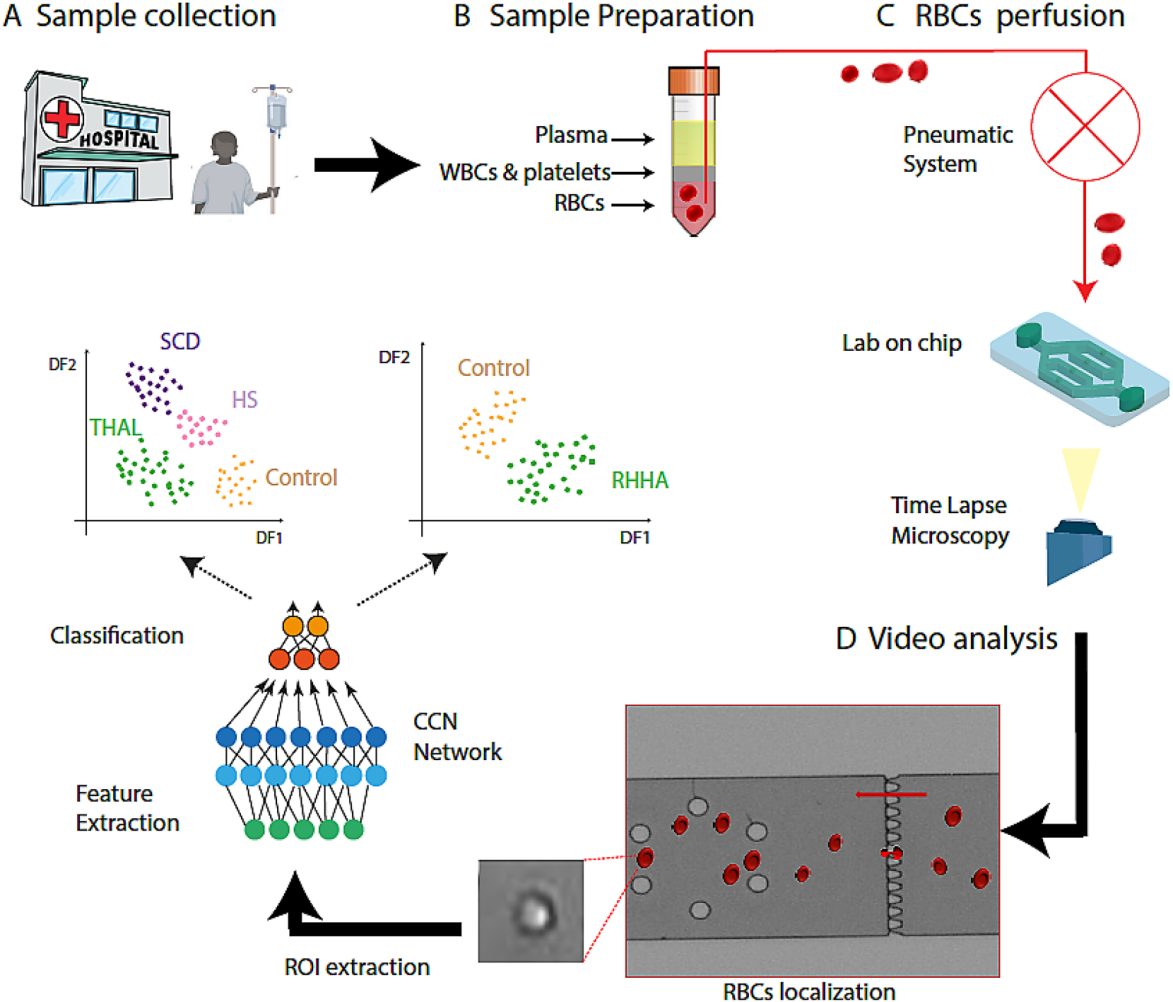
Scheme of the experimental workflow. (**A**) Sample collection (**B**) Sample preparation (**C**) RBCs perfusion (**D**) Video analysis.

Figure 1 shows the study’s workflow followed to perform these experiments: samples collection in the hospital, sample preparation, video recording under the microscope and video analysis.

### Samples collection

Human blood samples from 24 subjects (11 healthy donors, 4 SCD, 7 HS, and 2 THAL patients) were collected from University Hospital Vall d’Hebron for our study (Fig. 1A).

### Experimental process

#### RBCs separation and experimental solution

RBCs were isolated from the blood samples and diluted before perfusing them through the microfluidic device (Fig. 1B).

Considering that shear stress and changes in physiological solution can cause osmotic stress on RBC, rendering echinocytes or blocking visibility when recording videos^3,32^; we prepared different RBCs solutions and dilutions. We found that the optimal working solution for our experiments was physiological serum containing 1% BSA, 0, 25% of 0.25 M EDTA and 15% of glycerol see Supplementary Fig. S1A, Supplementary Information).

#### Microfluidic unit on a chip and RBCs perfusion

Optical inspection for experiments were performed using an optical Zeiss microscope and videos were recorded using a mono camera coupled to microscope. A Precision Pressure Control System was used to regulate the flow pressure in the microfluidic device. Videos were recorded for analysis while RBCs were perfused through the chip (Fig. 1C).

**Figure 2:**
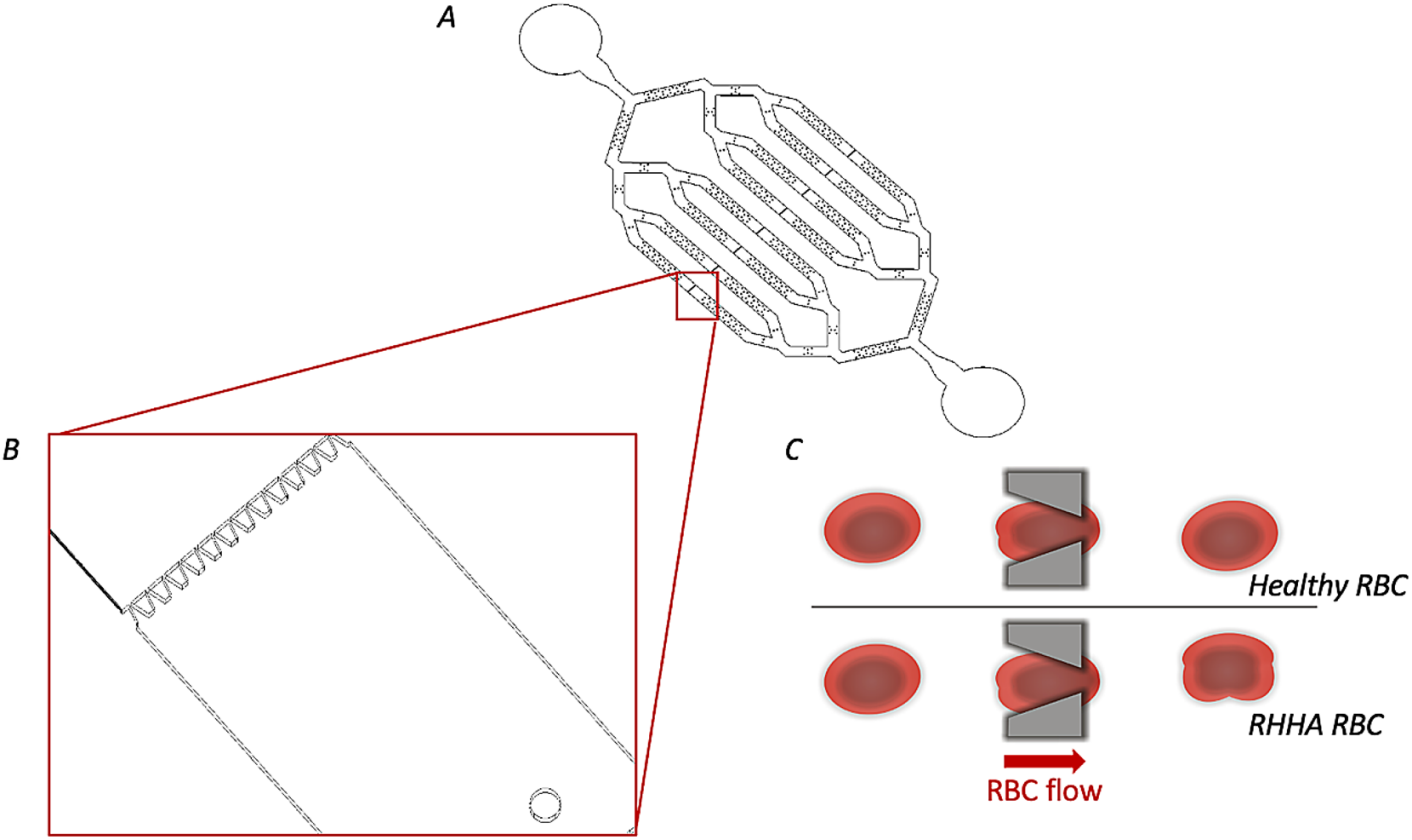
(**A**) Microfluidic unit on a chip designed in the study. It consists of a main channel branched until forming eight parallel microchannels. Each microchannels contain a row of filtering funnel-shaped micro-constriction to mimic the IES section of the spleen. (**B**) zoom out of filtering funnel-shaped constriction. (**C**) Representation of a healthy RBCs and a RHHA RBC passing through the microconstrictions. A healthy RBC deforms its shape and recovers it soon after passed the slits. On the contrary, in a RHHA patient the RBC capacity of returning to the original shape is compromise.

A microfluidic device was designed and fabricated with the aim of mimicking the filtering function of the red pulp’s spleen. It consisted of a main channel branched until forming eight parallel microchannels. Each microchannel contained a row of filtering funnel-shaped micro-constriction to mimic the IES section of the spleen. Due to their funnel shape, the distance between two slits varied from 1.5 to 6.8 μm. Of particular importance, the 1.5 μm narrowest distance defined to ensured RBC deformation when crossing a slit. Also, along each canal, there was a matrix of pillars (Fig. 2A,B). This matrix was designed to mimic the reticular mesh of the spleen. Total length for channel was 9.5 mm and height was 4.5 μm. Note that the height of the device was selected to limit RBC movement and maintain it in a planar orientation for a better visualization of its shape under the microscope when deforming itself while flowing through the micro-constrictions.

### Video analysis

Video analysis consisted of three steps: cell localization and automatic Region Of Interest (ROI) extraction, feature selection and data refining, and finally classification step (Fig. 1D).

#### Dataset collection and analysis of images from human RBCs

79 different videos were recorded and analyzed from the 32 subjects included in this study. Average duration of videos collected is 61 s (± 12 s) with an average number of frames of 1819 (± 483). Cells were automatically localized in each video frame, and a ROI around each cell was extracted and stored as a single data sample. This preliminary work did not include a cell tracking step, but rather each cell passing the barrier is pictured at different time point in order to collect groups of cell appearances for each category of disease and control.

The cell deforms its shape and recovers it soon after passed the slits in the case of a healthy RBC. On the contrary, when the RBC was from a RHHA patient, the RBC capacity of returning to the original shape is compromised (Fig. 2C). According to this assumption, we focused the analysis on the ROIs collected after the barrier.

Table 1 shows the number of ROIs for each anemia condition (control vs RHHA) after the slit barrier.

**Table 1:** Number of videos and ROIs analyzed in the study. The numbers in the table represent the number of videos recorded during the experiments and the number of ROIs extracted from the corresponding videos. Numbers are listed considering the total of the individuals included in the study, then divided in categories.

Sample	Number of VIDEO	Number of ROIs after the barrier
Total	79	3442
Control	30	1259
RHHA	49	2183
SCD	11	876
THAL	10	406
HS	28	901

*ROI* Region of interest, *RHHA* rare hereditary hemolytic anemia, *SCD* sickle cell disease, *THAL* thalassemia, *HS* hereditary spherocytosis.

Each extracted ROI was processed through a Deep Learning architecture (Fig. 1D) and coded into a list of numerical descriptors with the aim of assessing the lack of deformability. Due to the fact that we used a pretrained DL network, and no further fine tuning procedure is performed over the dataset acquired in this study, this procedure is called “transfer learning”.

We implemented Leave-One-Experiment-Out (LOEO) cross-validation procedure to assess the performance of the proposed methodology, so that on average the number ROIs used as training samples is 3398. Such a strategy was preferred to demonstrate the robustness of the approach, avoiding depending on different acquisition conditions (i.e. image illumination, channel pressure, RBC positioning of the chip). In this strategy, the ROIs extracted in a given video are left out for testing and the remaining ones acquired in the other videos are used for training the model. The procedure is then repeated exhaustively over all the 79 videos considered in the analysis.

We performed two different studies for the validation procedure: first, we calculated the accuracy of recognition of healthy (label 0) vs unhealthy (label 1) videos. Then, we also considered a more challenging scenario in which we tried to recognize individual kinds of anemia, by discriminating healthy (label 0) vs SCD (label 1), THAL (label 2), and HS (label 3) categories.

##### Two-class problem: healthy vs unhealthy

In this first study, results were initially collected in terms of classification accuracy at the single cell level and then in terms of the final label assigned to the entire experiment. Two distinct cooperative strategies are used for the task: majority voting and unhealthy percentage limit criteria.

The rationale was that even in the case of RBCs in RHHA not all RBCs show the same loss of deformability. Anyway, by applying a cooperative strategy procedure over the labels assigned to the cells in a given experiment, the approach allows understanding a global RBCs phenotype rather than an individual RBC behavior.

In addition, we also argue that RBC deformation persistence is exposed to temporal variation thus leading to the fact that in some frames the same cell appears as a “normal” cell while in some other frames it appears with a persistent loss of elasticity. Not less relevant is the fact that each frame represents the 2D view of a quasi-3D scene in which cells move in a 3D space and are visualized over a 2D domain with focus z plan automatically set by microscope. The projection errors may also contribute to the visual lack of persistence of the cell deformation.

The *majority voting* assigns to the video the most voted class among those assigned to the ROIs extracted. In the *unhealthy percentage limit*, the system assigns the unhealthy label (i.e., label = 1) if the percentage of ROIs assigned to an unhealthy label exceeds a predetermined limit value (e.g., 30%, 40%, etc.) over the entire video.

**Figure 3:**
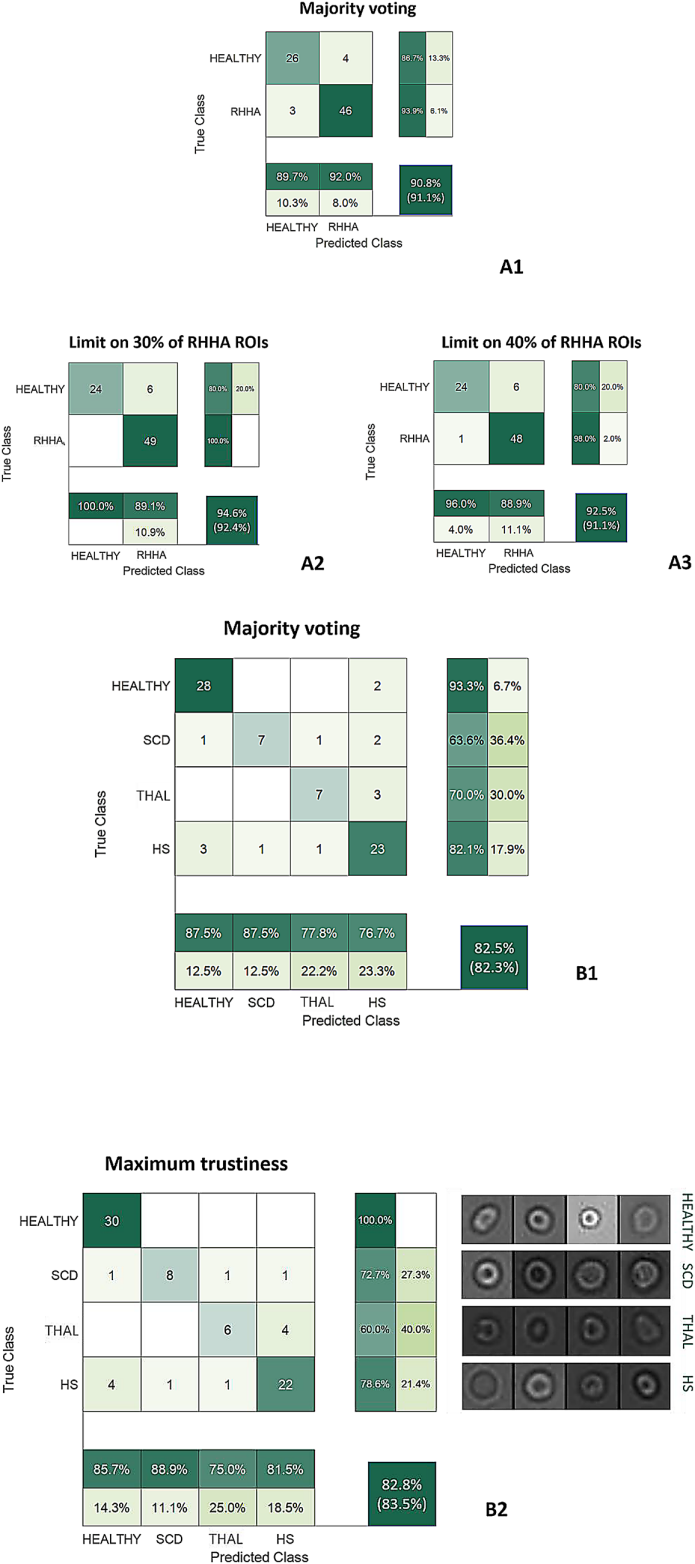
Confusion matrices. (**A1**) Confusion matrix reporting the results of the majority voting for the two-class problem. (**A2**–**A3**) Confusion matrices reporting the unhealthy percentage limit criteria for the two-class problem. (**B1**–**B2**) Confusion matrices reporting the results of the majority voting and of the maximum trustiness criteria for the four-class problem.

Figure 3A1–A3 report the accuracy results for the majority voting and the *unhealthy percentage limit* criteria for the two-class problem, considering two percentage limit values of 30% and 40%. Note that, using the 30% limit value we totally recognize RHHA subjects with no false-negative values.

More specifically, in the matrix in Fig. 3A1, out of 30 healthy experiments, the system recognized 26 as healthy and 4 as RHHA, and from 49 experiments from RHHA, it recognized 3 as healthy and 46 as RHHA. It means that after analyzing all the cells of the same experiments and taking the majority result (i.e., the most frequent class assigned) then the entire video was assigned to the majority class.

When moving to the matrix in Fig. 3A2–A3, final labels were assigned after a threshold value was used to filter out cells according to the percentage of cells belonging to the RHHA class in every single experiment (video). Therefore, in Fig. 3A2, we used a threshold value of 30% meaning that if more than 30% of the cell pictures tested in a given experiment were assigned to the RHHA class, then the entire experiment was assigned to the RHHA class. In Fig. 3A3, the percentage was increased up to 40%. In these two cases, out of the 30 healthy experiments, 24 were assigned as healthy and 6 were assigned to the RHHA class but all the 49 experiments from RHHA were recognized as unhealthy and only one was misclassified as healthy when using 40% of the minimum percentage of positive cells.

##### Four-class problem: healthy vs SCD, THAL, and HS

In the four-class problem, in addition to the majority voting cooperative strategy, we applied a *maximum trustiness criterion*. Given that the classification model provides a label and a score associated, in this second approach, the video is assigned to a certain class if the sum of the scores assigned to the ROIs of the same video to that class (out of the four considered) is the highest one. Figure 3B1–B2 illustrate the confusion matrices of the majority voting and of the maximum trustiness criteria, respectively.

More in detail, out of the 30 healthy videos, 28 were correctly assigned to healthy and only 2 were assigned to the HS class. Out of the 11 videos from the SCD class, 7 were correctly classified and 4 were misclassified over the three remaining classes. Out of the 10 videos from the THAL class, 7 were correctly classified and 3 were misclassified into the HS class. Out of the 28 videos from the HS class, 23 were correctly classified while 3 are misclassified as healthy, and 2 were equally assigned to SCD and THAL categories.

When passing to matrix in Fig. 3B2, results change according to the maximum sum of scores assigned to all the cell pictures of the same video. Therefore, all the 30 healthy videos were correctly assigned to healthy class. Out of the 11 videos from SCD, 8 were correctly assigned while 3 are equally misclassified among the remaining classes. Out of the 10 videos from the THAL class, 6 were correctly classified and only 4 were assigned to the HS class. Lastly, out of the 28 videos from the HS class, 22 were correctly classified and 6 were misclassified.

**Figure 4:**
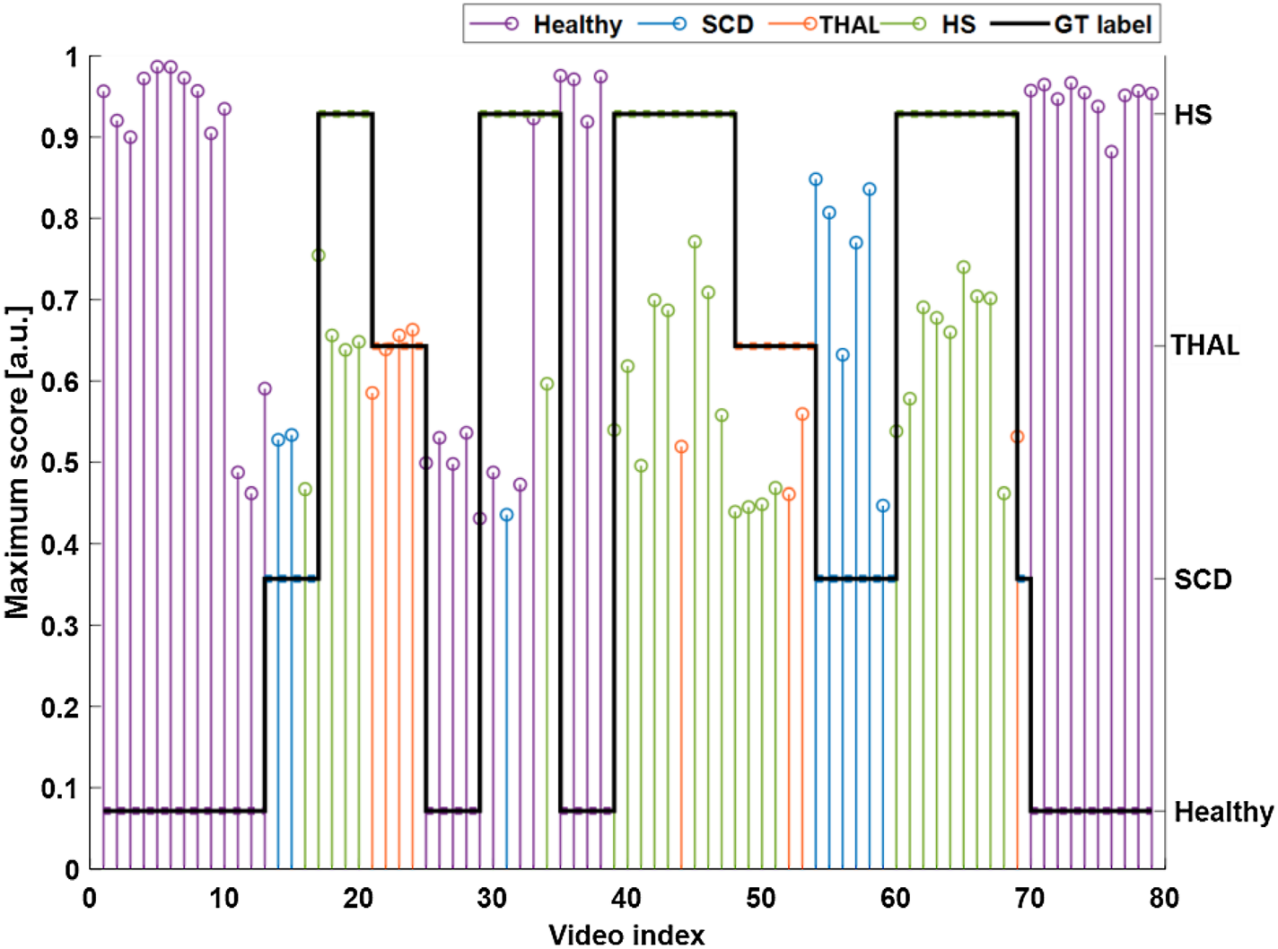
Sketch of the scores assigned to each of the 79 videos and related ground truth label. The height of the vertical bars represents the maximum score obtained for the assigned class; Colors indicate the category assigned according to the legend to the top-right corner. The black solid stair line indicates the expected category for each video as indicated by the right y-axis labels. As it can be observed, the healthy class (purple lines) are very well recognized and the related normalized score is very high, indicating that the scores of the unassigned class were very low. The visual results also confirm the fact that there are no false negative healthy subjects (i.e., a subject with a disease assigned to the healthy category) as also represented in the confusion matrix. Regarding the three anemia conditions, the values of the scores are smaller indicating the critical task to solve, but also in this case, there are a very few errors of classification, mostly due to the misclassification between THAL and HS samples (e.g., video n. 44 should be HS and instead is assigned to THAL, and videos n. 48–51 that should be THAL and are assigned to HS).

To fully understand the role of the scores and evidence the potential of the method, we also show a sketch of the scores assigned to each of the 79 videos and related ground truth label in Fig. 4.

The height of the vertical bars indicates the score values assigned to each video (video index on the x-axis). Colors indicate the category assigned according to the legend to the top-right corner. The black solid stair line indicates the expected category for each video as indicated by the right y-axis labels.

Note that, for controls, not only the scores were generally higher (the range of the score is [0, 1]) but also there are not any healthy videos incorrectly assigned to a different category. Regarding the three anemia conditions, the values of the scores are smaller indicating the critical task to solve, but also in this case, there are a very few errors of classification, mostly due to the misclassification between THAL and HS samples (Fig. 4).

## Discussion

We present a methodology that combines the use of a microfluidic unit with machine learning data analysis from video recorded images for evaluating the premature lack of plasticity of RBCs in patients with RHHA, through an objective and reliable platform, a better characterization of RBCs in RHHA through machine learning algorithms will enable the stratification of patients based on severity and/or response to treatment. Thus, facilitating personalized medicine and development of new treatment strategies. Beyond standard decision system implementation (e.g., single cell classification model) the considered strategies use two additional cooperative learning approaches. In the first one, i.e., the majority voting scheme, the most voted label over all the cell images from the same video is finally assigned to the entire video. In a second strategy, the maximum sum of scores assigned by the classifier to each class is finally used to decide the maximally scored class to assign. These strategies allow us overturning the problem of so-called false instances (diseased cells detected as healthy cells and viceversa) leading to focus on unhealthy patient more than to single unhealthy cells. This approach starts from two main assumptions: the former is that not all cells belonging to a patient are necessarily unhealthy; the latter is that healthy cells can be appeared unhealthy due to the 2D projection errors of a 3D scene.

Previous work from our group described a functional spleen on a chip unit, incorporating the fast and slow spleen’s compartments^22^. Mimicking the spleen function, only 10% of RBCs circulates through the slow compartment which involves the filtering function, as also it is estimated to occur in the spleen. We designed a microfluidic unit to process a higher number of RBCs mimicking the conditions experienced by human RBCs while passing through the microcapillaries of the red pulp in the spleen, which microcirculatory structures have a dimension from 1 to 3 μm.

The RBC can show two kinds of motions in a shear flow when suspended in a blood plasma-like medium. The RBC acts as a rigid cell with a flipping motion under flow in low viscosity condition. Increasing the shear rate, the RBC acquires a rolling behavior on movement, rotating into an orbit with the axis of symmetry perpendicular to the shear plane^33^. Then, the height of the designed microchannels aims to maintain these RBCs in a planar orientation to ensure that we can observe the deformation of their larger side when passing through the slits. Slits present a distance of 3 µm in the narrowest part, essential to achieve our goal of mimicking the microcirculatory behave when the RBC deforms itself while flowing into the device.

To assess our microfluidic unit on a chip as a valid device for the characterization of the RBCs, analysis was made using control and RHHA samples. RHHA subgroups were composed by SCD, THAL and HS, conditions in which, due to their structural defects, RBCs lose the ability to deform themselves and flew through the capillaries in the spleen. As also described in other studies, the loss of deformability can be mostly observed after the RBC passing through a microconstriction^2^. Therefore, our hypothesis for analysis was that RBCs from patients and RBCs from controls should have different performances when passing through the slits. The variability of the RHHA disease and related morphological manifestation in RBCs shape are also accounted by considering a high number of human samples (79 videos from 32 different subjects) and related cell images (more than 600 K).

The results show the capability of our system not only to distinguish between healthy controls and patients with an average efficiency of 91%, but also to distinguish between RHHA subtypes, with an efficiency of 82%, proving the possibility of using our platform for the characterization of the RBCs in RHHA disorders. With the maximum trustiness criterion, the number of false positives vanishes, and all the healthy subjects are perfectly recognized. As expected, the more challenging scenario exhibits lower performance with respect to the two-class problem, but it is possible to differentiate among the different RHHA.

Concerning the presence of false negatives, some factors should be taken into account as possible cause. Among others, a mild phenotype of the disease or patients under transfusion therapy as RBCs from patients are mixed with RBCs from donors. Then, not all RBCs in a RHHA patients are “unhealthy”, and not all RBCs in a healthy control are “healthy”, the “healthy” and “unhealthy” status is primarily age dependent, meaning that old RBCs will be more likely to be labeled as “unhealthy” by the platform.

Thresholds can be established for labeling a sample as “healthy” or “unhealthy” within the majority voting approach. The establishment of a specific threshold would depend on the use of the platform for patient screening or for disease evolution monitoring. For example, using a majority voting with a 30% *unhealthy percentage limit* criteria the platform shows high sensitivity, recognizing all RHHA subjects with no false negative value and only 10.9% of the healthy subjects were recognized as false positive (6 of 30 individuals). In this way, no patients will be excluded.

The ability of having results based on single ROIs (cells) represents an advantage of our platform, something not possible at the moment using other techniques in the market, where the results obtained corresponds to an average of sample’s cells. This competence can have an interesting impact on the development of new techniques for the understanding of the complexity of the disease or of the heterogeneity, among patients, in response to treatment. At the moment, such monitoring techniques based on assessment of single cells are not implemented, on the contrary of other techniques for RHHA diagnosis, among these EMA binding test and Ektacytometry for HS, and Hb’s fraction for hemoglobinopathies. Indeed, choosing the proper treatment is becoming more challenging due to the recent advances in the molecular basis underlying RHHAs which have led to many new drugs and gene therapy approaches that are still in clinical trials.

The percentage of abnormal RBCs in a patient sample could be used as a parameter of disease’s evolution or of response to treatment to be monitored over time.

We may be able to observe the ongoing variations in the percentage of cells with lost ability of changing shape and to correlate this information with the clinical picture of the patients, allowing their stratification according to disease severity or prediction of acute event in SCD.

## Conclusions

The approach of combining microfluidic platform together with deep learning described in this study constitutes an interesting tool for the study of RBCs disorders.

A low performing camera can be used with the aim to acquire a large field of view, in which many RBCs can be visualized simultaneously in the same frame. The relatively low spatial resolution achieved on each cell is compensated by the high performing image analysis module and by the flow control system. Furthermore, the use of deep learning analysis helps to overcome the difficulty faced when working with high-speed movies, such as unavailable or unaffordable recording using a speed camera.

The results presented here indicates that our platform is a valid tool for discriminating RHHA patients from healthy donors, with an efficiency of 91%, and among specific disorders with an efficiency of 82%. Nevertheless, further studies to improve sensitivity and increase robustness of the results are needed to validate the clinical use of these microfluidic platforms.

The characterization of RBCs rheological behavior opens the possibility of application for patient's stratification according to disease severity and response to treatments and consequently it would open to the chance of personalized treatment.

This platform could be beneficious also in the biobank field for the optimization of protocol for blood storage or stratification of donors or for other disease models, such as cardiovascular diseases.

## Methods

### Experimental process

#### Samples collection

The study was conducted in accordance with the Declaration of Helsinki and was approved by the Vall d’Hebron University Hospital Ethics Committee (n.367 and n.464 CPMP/ICH/135/95). Informed consent for blood samples collection was obtained from participants.

Experiments were performed collecting on ethylenediaminetetraacetic acid (EDTA) peripheral blood samples from healthy donors, SCD patients, HS patients, Thalassemia. Samples were stored at + 4 °C and analyzed within 48 h after their collection.

For the normal control, subjects with normal complete blood count (CBC) parameters for anemia were taken.

#### RBCs separation and experimental solution

Packed RBCs were isolated from whole blood. RBCs count from CBC parameters were considered to calculate the volume of whole blood needed in order to set the number of RBCs to a same value for each experiment. The correspondent amount of whole blood was centrifuged at 0.4 rcf for 5 min at + 4 °C. After removing buffy coat and plasma, packed RBCs were suspended and washed in physiological serum (0.9% NaCl).

After centrifugation, supernatant was removed, and packed RBCs re-suspended in 1 mL of physiological serum. Our working solution consisted of physiological serum containing 1% BSA, 0.25% of 0.25 M EDTA and 15% of glycerol (Sigma Aldrich). Finally, working solution containing 1% of re-suspended packed RBCs was prepared.

#### Microfluidic unit on a chip fabrication

The device was fabricated using standard photolithography and soft lithography techniques. A wafer was first cleaned in three consecutive solvent baths of acetone, isopropanol and water. Then, after dehydration at 150 °C a precise multistep procedure was performed in order to obtain the desire thickness for the microfluidic device. (1) A layer of SU8-2005 was spin coated above the silicon wafer through two steps (first 15 s at 500 with an acceleration of 100 rpm/s and second 30 s at 8000 rpm with an acceleration of 300 rpm/s). Then, wafer was soft baked through three steps of temperature (1 min at 65 °C, 2 min at 95 °C and 1 min at 65 °C) and exposed to UV light (8,03 mJ/cm^2^). Finally, it was post-baked through three steps of temperature (2 min at 65 °C, 3 min at 95 °C and 1 min at 65° and developed using SU-8 developer for 30 s. Dimensions of the channels were verified by microscopy and profilometer measurements. The microfluidic device replicas were made of PDMS prepolymer mixture (Sylgard184, Dow Corning), a silicone elastomer diluted 10:1 with a cross linker using microfabrication and molding techniques previously described^22^. After polymerization, PDMS was peeled off and inlet and outlet holes made using a 1 μm puncher.

PDMS and glass slides were exposed to O_2_ plasma, immediately pressed together and heated at 95 °C for 10 min to form a permanent bond.

#### RBCs perfusion

The experimental set-up consisted of a Precision Pressure Control System (P2CS, Biophysical Tools GMBH) to regulate the flow pressure in the microfluidic device. 1 mm flexible plastic tubing (Tygon) at the inlet hole connected pump and microfluidic chip.

Experiments were performed at room temperature at a constant pressure of 100–150 mBar. Before recording the videos, RBCs were left flowing for 1 min to stabilize the microfluidic unit conditions. Approximately 1000 frames for each video were recorded and camera speed was 85 fps.

Optical inspection for experiments were performed using a microscope (Zeiss) and an axiocam 503 mono camera. While RBCs perfusion through a filtering unit on a chip, several videos were recorded for analysis.

### Video preprocessing

Each video was cropped to extract a limited area of interest around the slit barrier. This procedure assisted in eliminating confounding structures that may alter the automatic cell localization result. This step was also performed to face the problem of cell velocity variability around the barrier due to unpredictable change in the flux. In fact, we observed that when an RBC cell stops in the barrier, fluid dynamic phenomena arise for which the other cells are slowed down. On the contrary, when a cell restarts opening a gap in the slit barrier it creates a suction effect that accelerates the arriving. Cell velocity variability makes the cell appearance changing over time in an unpredictable manner. As a consequence, in some cases, cells disappear or go out of focus.

### Video analysis

#### Cell localization and automatic ROI extraction

RBCs are localized through the Circular Hough Transform, an algorithm specifically designed to localize almost circular object in a given scene^34^. This algorithm has already demonstrated to be efficient in localizing cancer cells and immune cells in previous work^35–37^. A ROI of fixed size is extracted around each localized cell (Fig. 1D), frame by frame, by cropping the frame in a square region around the center of the cell. In this way, we collected two sets of ROIs. ROIs whose cells appear before the barrier (ROI-before) and ROIs corresponding to cells after the barrier (ROI-after). Our rationale was that RBCs in patients with anemia exhibit loss of deformability properties. Due to this, in patients with anemia, RBCs crossing the barrier cannot rapidly reset their shape. The comparative analysis of ROI-after the slit barrier taken from control subjects and patients could evidence such assumption.

#### Deep learning analysis and feature extraction

Multivariate analysis is even more crucial for recognize patterns in a given dataset and to provide proof of concepts to biological phenomena. However, an even more critical step is the extraction of specific features to characterize object shape, morphology, and movement variation. The advent of Deep Learning (DL) CNN architecture has opened new possibilities for the image and video analysis community^38^. In fact, DL allows analyzing an image as it appears and to transform it into a vector of low-level features automatically extracted through the internal layers of the architecture. Such approach is called “transfer learning” since it allows to transfer the result of a learning procedure elsewhere performed on very different datasets of images (e.g., so-called pre-trained DL networks) into new images, by exploiting the ability of the network to extract the relevant information of a given image in the form of numerical descriptors^39^. In this way, by applying the well-known AlexNET DL network^40^, we transformed each ROI extracted after the slit barrier into a vector of features for further pattern recognition analysis. No training phase is therefore required in this step, due to the capability of the network to code each ROI into numbers according to an already robust pretraining procedure.

#### Feature selection

The features extracted from the internal layers of AlexNET DL network (here we used the pool5 layer that corresponded to the 3rd Max Pooling Layer), had to be preliminarily processed. In fact, the high number of information the network provided contained some redundant features that commonly are related to the background of the images (i.e., the uniform, gray-shaded areas in the ROI) or to image characteristics not related to the cell morphological changes. Such useless information should present a strong uniformity over the different ROIs in the same video sequence since they are not related to RBC shape. For this reason, a preliminary unsupervised automatic feature selection procedure was applied with the aim of eliminating features exhibiting a small variation over the entire dataset under a given limit value. The procedure reduced the number of features of a factor of 50.

##### RBC classification set-up for healthy vs unhealthy

Support Vector Machine (SVM) classifiers with linear kernel had been used for the scope of constructing a classification model able to discriminate experiments with RBCs from control subjects and RBCs of patients with RHHA^41^. A leave one-experiment-out (LOEO) was used as cross-validation of the method in order to obtain experiment-independent, i.e., more general results.

Single cells were labelled as healthy (0) or unhealthy (1). However, the assumption was that not all RBCs cells exhibit an equal lack of deformability properties. To evidence this assumption, we also applied two kinds of cooperative learning approach: first, we applied the majority voting procedure over the labels assigned to the cells of the same experiment; second, we considered the opportunity to assign an unhealthy label to an experiment that exhibits a percentage of unhealthy cells larger than a given limit value (i.e., 30%, 40%, or larger).

##### RBC classification set-up for healthy vs SCD, THAL, and HS

With the aim to test the platform to the recognition of three distinct kinds of anemia, SCD, THAL, and HS, from control subjects, we also developed a specific set-up for the four-classes problem associated. The selected classification model was a four-classes SVM trained through a one-vs-all strategy. At each cell was then assigned a normalized score to belong to each class. In the standard approach, the class with the highest score was then selected for that cell. With the aim to perform cooperative learning, we also applied majority voting over the entire experiment and also a maximum-trustiness criterion. In the latter rule, all the scores to belong to the same class over the entire experiments were summed up. The class with the highest sum of score was finally assigned to the experiment.

## Supplementary Information

Supplementary Figure S1.

## Data Availability

The videos generated during and/or analyzed during the current study are available from the corresponding author on reasonable request.
