# Supplementary material for: Combining microfluidics with machine learning algorithms for RBC classification in rare hereditary hemolytic anemia
Source: Sci Rep. 2021 Jun 30;11:13553. doi: 10.1038/s41598-021-92747-2 (PMC8245545; doi:10.1038/s41598-021-92747-2)
Supplement: Supplementary file 1 — Supplementary Figure S1. [file 41598_2021_92747_MOESM1_ESM.pdf]

Combining microfluidics with machine learning algorithms for RBC classification in rare hereditary hemolytic anemia

Valeria Rizzuto, Arianna Mencattini, B. Álvarez-González, Davide Di Giuseppe, Eugenio Martinelli, David Beneitez-Pastor, Maria del Mar Mañú-Pereira, Maria José Lopez-Martinez*, Josep Samitier

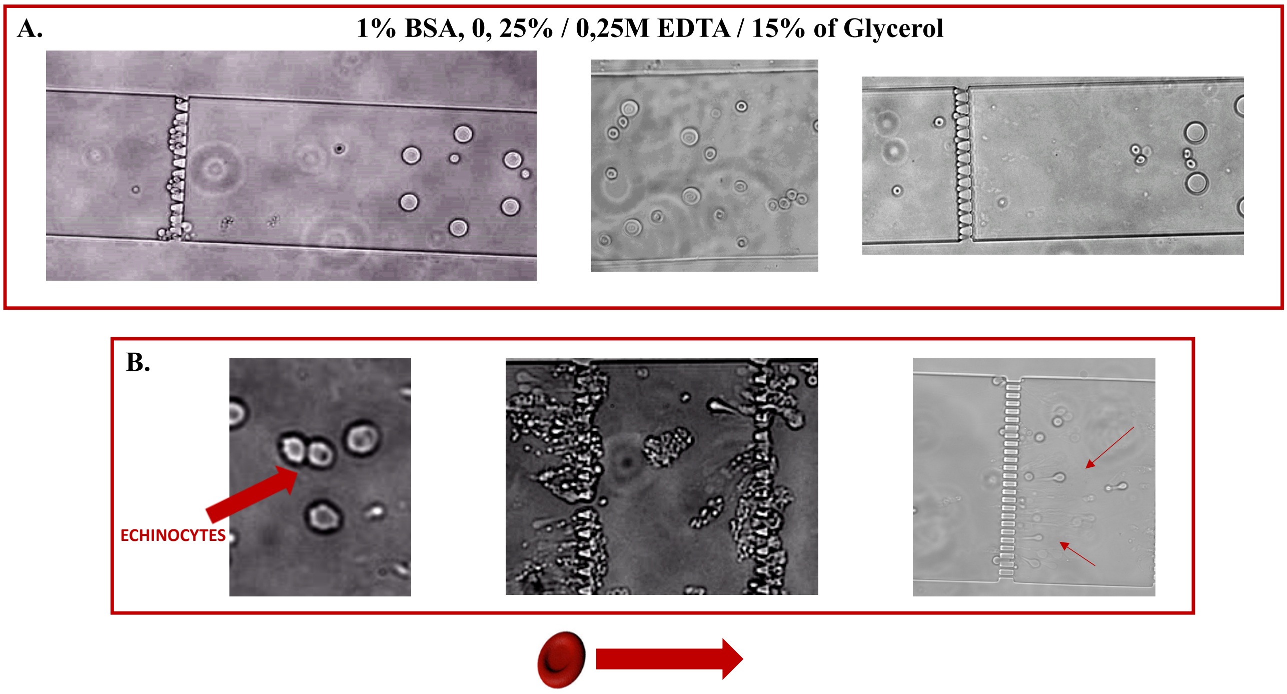

Figure S1 (Various reagents were tried and tested to get the optimal solution for the experiments. The main problems to be overcome is the formation of echinocytes but also of RBCs aggregates, especially after passing through the slits. Solutions tested during the optimization process include PBS / EDTA, PBS / EDTA / BSA 0.1%, Physiological saline solution / EDTA / BSA 0.1%, Physiological saline solution / EDTA, Physiological saline solution / EDTA / Poloxamer. A. With our final solution, containing BSA/EDTA/glycerol, our chip is clean with no RBC aggregations and/or echinocytes. B. With the other solutions it was not possible to carry out experiments while maintaining the physiological conditions of the RBCs.

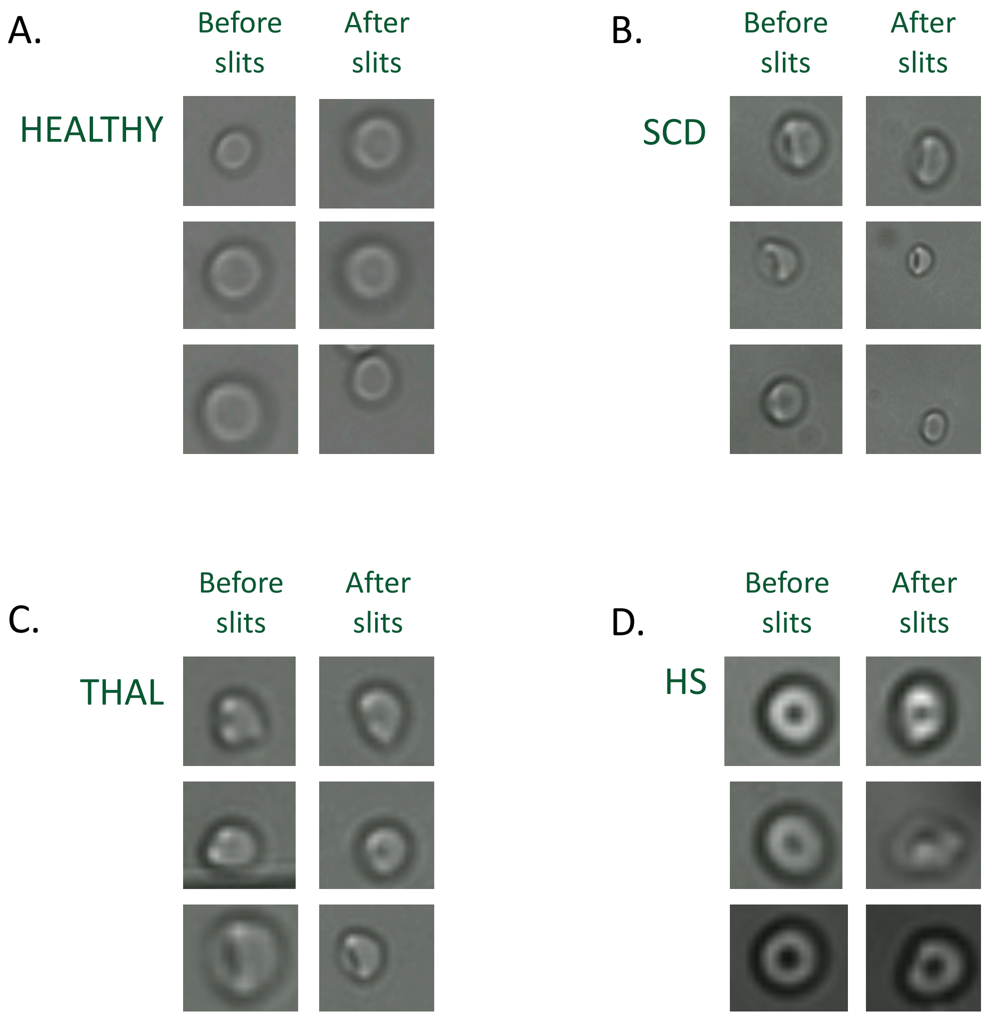

Figure S2 (Images of RBCs before and after passing through the microconstrictions. A. RBCs of healthy donors. B. RBCs from SCD patients. C. RBCs from THAL patients. D. RBCs from HS patients)
